# GREM1 is a novel serum diagnostic marker and potential therapeutic target for pancreatic ductal adenocarcinoma

**DOI:** 10.3389/fonc.2022.968610

**Published:** 2022-08-26

**Authors:** Sen Yang, Yalu Zhang, Yuze Hua, Ming Cui, Mengyi Wang, Junyi Gao, Qiaofei Liu, Quan Liao

**Affiliations:** ^1^ Department of General Surgery, State Key Laboratory of Complex Severe and Rare Diseases, Peking Union Medical College Hospital, Chinese Academy of Medical Science and Peking Union Medical College, Beijing, China; ^2^ Department of General Surgery, Anhui Provincial Hospital, The First Affiliated Hospital of USTC, Division of Life Science and Medicine, University of Science and Technology of China, Hefei, China

**Keywords:** gremlin 1 (GREM1), pancreatic adenocarcinoma (PDAC), tumor microenvironment, diagnosis, marker

## Abstract

**Objective:**

Pancreatic ductal adenocarcinoma (PDAC) is a highly malignant neoplasm with rising incidence worldwide. Gremlin 1 (GREM1), a regulator of bone morphogenetic protein (BMP) signaling, fine-tunes extensive biological processes, including organ morphology, cellular metabolism, and multiple pathological developments. The roles of GREM1 in PDAC remain unknown.

**Methods:**

Varieties of public databases and online software were employed to analyze the expressions at transcription and protein levels of GREM1 in multiple malignant neoplasms including PDAC, and in addition, its potential pro-tumoral functions in PDAC were further evaluated. A total of 340 serum samples of pancreatic disease, including PDAC, low-grade malignant pancreatic neoplasm, benign pancreatic neoplasm, pancreatitis, and 132 healthy controls, were collected to detect GREM1. The roles of serum GREM1 in the diagnosis and prediction of survival of PDAC after radical resection were also analyzed.

**Results:**

Bioinformatics analyses revealed that GREM1 was overexpressed in PDAC and predicted a poorer survival in PDAC. A higher protein level of GREM1 in PDAC correlated with stroma formation and immunosuppression by recruiting varieties of immunosuppressive cells, including T regulatory cells (Tregs), M2 macrophages, myeloid-derived suppressor cells (MDSCs), and exhaustion T cells into the tumor microenvironment. A higher level of serum GREM1 was observed in PDAC patients, compared to healthy control (*p* < 0.001). Serum GREM1 had a good diagnostic value (area under the curve (AUC) = 0.718, *p* < 0.001), and its combination with carbohydrate antigen 199 (CA199) achieved a better diagnostic efficacy (AUC = 0.914, *p* < 0.001), compared to CA199 alone. The cutoff value was calculated by receiver operating characteristic (ROC) analysis, and PDAC patients were divided into two groups of low and high GREM1. Logistic analyses showed serum GREM1 positively correlated with tumor size (hazard ratio (HR) = 7.097, *p* = 0.032) and histopathological grades (HR = 2.898, *p* = 0.014). High-level serum GREM1 (1,117.8 pg/ml) showed a shorter postoperative survival (*p* = 0.0394).

**Conclusion:**

Higher intra-tumoral expression of GREM1 in PDAC contributes to tumor stroma and immunosuppressive tumor microenvironment, presenting its therapeutic potential. High-level serum GREM1 predicts poorer survival after resection. A combination of serum CA199 and GREM1 shows a stronger diagnostic efficacy in PDAC.

## Introduction

Pancreatic cancer, mainly referred to as pancreatic ductal adenocarcinoma (PDAC), is the fourth leading fatal neoplasm, with a deteriorating tendency in the next decade (1). The relatively low incidence is coupled with disproportionately high mortality, with an average annual incidence rate of 12.5 per 100,000 population in the United States but with a 5-year survival rate of approximately 10% (2). Surgical therapy is currently the only curable approach. However, most patients with PDAC are diagnosed with advanced stage, due to the predicament of early diagnosis, and most patients relapse after surgical treatment (3). Furthermore, the median survival of patients with metastatic disease is only 3 months (4, 5). PDAC is featured as the conspicuous chronic inflammation, where massive inflammatory signaling cascade and abundant immune cells occur. Chaos in external environmental signals is comprehensively advantageous to tumor cell survival and proliferation (1). Furthermore, the inactivation of antitumor immunity and prevalence of pro-tumor immunity symbolize the pancreatic tumor microenvironment, leaving PDAC as one of the refractory malignant diseases (6). All in all, early diagnosis and effective therapeutic methods are urgently needed in clinical practice. The most commonly used serum marker to diagnose PDAC is carbohydrate antigen 199 (CA199). However, nearly 30% of the PDAC patients have a normal level of CA199; in addition, in the early stage of PDAC, the positive rate of CA199 was even lower; therefore, novel markers that could improve the diagnostic roles of CA199 are urgently needed (7).

The bone morphogenetic protein (BMP) was firstly reported by Marshall Urist in 1965, which is a demineralized bone matrix with significant osteoinductive activity (8). As the accumulation of research advances, the formidable signaling has revealed its participation beyond osteogenesis and bone remodeling, in multiple biological processes such as embryonic development, angiogenesis, iron metabolism, inflammation, and sexual reproduction (9). BMPs belong to the transforming growth factor β (TGF-β) family, delivering signals *via* type I and type II serine–threonine kinase receptors and intracellular downstream effectors. Furthermore, BMP signals are fine-tuned by various agonists and antagonists (10). GREM1, as an antagonist, is predominantly expressed in stromal cells and encodes the generation of the secreted glycosylated protein to combine with BMP-2, BMP-4, and BMP-7 to typically form homo- and heterodimers, binding to selective BMPs to prevent ligand–receptor interactions and subsequent downstream signaling (11). Several studies have reported the overexpression of GREM1 by cancer-related stromal cells, promoting tumor cell proliferation, which suggests that GREM1 is responsible for the specialized tumor microenvironment (12). GREM1 enhances the TGF-β-mediated epithelial-to-mesenchymal transition (EMT) as the result of BMP maintaining epithelial integrity by antagonizing TGF-β. Moreover, GREM1 production by cancer-associated fibroblasts (CAFs) expedited the fibrogenic activation and facilitated breast cancer cell intravasation and extravasation in co-injection xenograft zebrafish models (13).

As far as we know, the roles of GREM1 in PDAC remain unknown. Considering its regulatory roles in tumor stroma and inflammatory cells, in this study, the different expression levels at transcription and protein levels and potential pro-tumoral roles were evaluated by using varieties of online public databases. Further, the diagnostic and predictive roles of serum GREM1 were analyzed by using a large cohort of patients.

## Materials and methods

### Ethics statement

The study was approved by the Ethics Committee of Peking Union Medical College Hospital. All patients enrolled in our study provided written informed consent for the scientific research use of the samples.

### Patients and serum samples

Serum from 340 patients and 132 healthy controls (HCs) were collected from the Clinical Biobank of Medical Science Research Center of Peking Union Medical College Hospital, including 128 cases of PDAC, 39 cases of intraductal papillary mucinous neoplasm (IPMN), 47 cases of pancreatic solid pseudopapillary neoplasm (SPN), 54 cases of pancreatic neuroendocrine tumor (pNET), 31 cases of serous cystadenoma (SCN), 26 cases of mucinous cystadenoma (MCN), and 15 cases of chronic pancreatitis (CP) and pancreatic pseudocyst (PPC). The characteristics of patients are summarized in **Table 1**. A total of 117 patients receiving radical resection operations, from September 2013 to December 2017, were conducted in a follow-up cohort, with the end time up to 19 November 2019, and ultimately 82 patients acquired the survival status and overall time. The male-to-female ratio was 72:56. The age of the patients ranges from 36 to 79 years with a mean age of 61.0 ± 9.1 years old and a median age of 62 years. Of the 82 cases, 52 patients died, and 30 were alive to the end time of follow-up. The median survival time of follow-up was 512 days. The diagnosis and staging were based on the 8th edition of the American Joint Committee on Cancer (AJCC). Inclusion criteria were as follows: 1) older than 18 years, 2) pathologically diagnosed with PDAC, and 3) receiving radical operation. Exclusion criteria were as follows: 1) pathological specimens could not be obtained and 2) refused follow-up.

**Table 1:** Basic characteristics of 340 patients with pancreatic diseases.

	PDAC	IPMN	SPN	pNET	SCN	MCN	CP+PPC
Total number	128	39	47	54	31	26	15
Age (years)	61.0 ± 9.1	59.3 ± 10.6	31.6 ± 10.4	50.1 ± 11.1	54.5 ± 13.1	47.5 ± 13.4	51.9 ± 15.2
Gender							
Male	56 (43.8%)	16 (41%)	35 (74.5%)	32 (59.3%)	25 (80.6%)	23 (88.5%)	3 (20%)
Female	72 (56.2%)	23 (59%)	12 (25.5%)	22 (40.7%)	6 (19.4%)	3 (11.5%)	12 (80%)
Surgery							
TPS	3 (2.3%)	6 (15.4%)	0 (0)	1 (1.9%)	0 (0)	0 (0)	0 (0)
PD	62 (48.4%)	14 (35.9%)	5 (10.6%)	6 (11.1%)	7 (22.6%)	2 (7.7%)	4 (26.7%)
PPPD	6 (4.7%)	6 (15.4%)	0 (0)	4 (7.4%)	3 (9.7%)	0 (0)	1 (6.7%)
DP	2 (1.6%)	3 (7.7%)	14 (29.8%)	5 (9.3%)	7 (22.6%)	9 (34.6%)	1 (6.7%)
DPS	41 (32%)	6 (15.4%)	13 (27.7%)	17 (31.5%)	6 (19.4%)	13 (50%)	3 (20%)
LR	1 (0.8%)	2 (5.1%)	15 (31.9%)	21 (38.9%)	8 (25.8%)	2 (7.7%)	6 (40%)
Biopsy	13 (10.2%)	2 (5.1%)	0 (0)	0 (0)	0 (0)	0 (0)	0 (0)
Tumor location							
Head and neck	86 (67.2%)	29 (74.4%)	22 (46.8%)	29 (53.7%)	19 (61.3%)	5 (19.2%)	9 (60%)
Body and tail	42 (32.8%)	10 (25.6%)	25 (53.2%)	25 (46.3%)	12 (38.7%)	21 (80.8%)	6 (40%)
Neoadjuvant chemotherapy	2 (1.6%))	0 (0)	0 (0)	0 (0)	0 (0)	0 (0)	0 (0)

PDAC, pancreatic ductal adenocarcinoma; IPMN, intraductal papillary mucinous neoplasm; SPN, solid pseudopapillary neoplasm; pNET, pancreatic neuroendocrine tumor; SCN, serous cystadenoma; MCN, mucinous cystadenoma; CP, chronic pancreatitis; PPC, pancreatic pseudocyst; TPS, Total pancreatectomy and splenectomy; PD, Pancreaticoduodenectomy; PPPD, Pylorus-preserving pancreaticoduodenectomy; DP, Distal pancreatectomy; DPS, Distal pancreatectomy and slenectomy; LR, Local rescetion.

### Detection of serum GREM1 levels

Serum samples from 340 patients and 132 healthy people were collected and underwent enzyme-linked immunosorbent assay (ELISA). Serum GREM1 levels were quantitatively detected using a GREM1 ELISA Kit (MM-60567H1, Meimian, Jiangsu, China), according to the manufacturer’s protocol.

### Gene expression profiling interactive analysis

Gene Expression Profiling Interactive Analysis (GEPIA) (http://gepia.cancer-pku.cn/) is an online web tool that explores cancer and normal gene expression profiling and interactive analyses based on The Cancer Genome Atlas (TCGA) and Genotype-Tissue Expression (GTEx) databases (14). With the use of the GEPIA database, the gene expression profile of GREM1 was analyzed across 33 tumor samples and paired with normal tissues by the module ‘Expression DIY’, selecting ANOVA as the differential method. The prognostic values, overall survival (OS), and disease-free survival (DFS) of GREM1 were analyzed by using the function module ‘Survival Plot’ in several tumor types, and the ‘Median’ was arranged as the ‘Group Cutoff’. The correlation function was used to predict the influence of the GREM1 expression on the genes of the T-cell exhaustion state, selecting the ‘Spearman’ as the ‘correlation coefficient’.

### Oncomine

Oncomine (https://www.oncomine.org/) is a gene chip-based database that contains substantial tumor microarray datasets (15). The log2-transformed form was utilized to represent the transcriptional levels of GREM1, and the inclusion criteria were designed as the ‘Fold change > 2’ and ‘*p*-value < 0.05’.

### The human protein atlas

The Human Protein Atlas (HPA) database (https://www.proteinatlas.org/) is an interactive data-mining platform, integrating substantial distribution information of human protein from more than 20 kinds of cancer at the cellular and histopathological levels. The immunohistochemical (IHC) staining images of GREM1 in normal pancreas tissue and PDAC, the protein expression, and the survival plot were all collected from the HPA database.

### Kaplan-Meier plotter

Kaplan-Meier plotter (http://kmplot.com/) is a potent public online database for survival analysis of 21 tumor types in the basement of substantial RNA-seq and next-generation sequencing. The OS of GREM1 under the different conditions of immune cell infiltration was evaluated by the Kaplan-Meier plotter database. Hazard ratio (HR) and 95% confidence interval (CI) were calculated automatically according to ‘Auto select best cutoff’.

### LinkedOmics

LinkedOmics (http://www.linkedomics.org/) is an available web portal for users to analyze multi-omics data on the basement of 32 cancer types (16). Functional enrichment and prediction of GREM1 were performed using gene ontology (GO) and Kyoto Encyclopedia of Genes and Genomes (KEGG) pathway analysis from the database, containing biological process (BP), cellular component (CC), and molecular function (MF).

### GeneMANIA

GeneMANIA (http://www.genemania.org) is a potent and convenient website tool to predict gene function, analyze gene lists, and perform functional assays (17). We used this tool to manufacture the gene–gene interaction network of GREM1 and the top 20 correlated genes.

### STRING

STRING (https://www.string-db.org/) is a useful database predicting protein–protein interactions by physical and functional association. A protein–protein interaction (PPI) network of GREM1 was implemented to scan correlated genes for GREM1 function prediction.

### TISIDB

TISIDB (http://cis.hku.hk/TISIDB/index.php) is a public portal for tumor and immune system interaction, as well as the integration of numerous heterogeneous data types (18). Detailed analysis of immune infiltration of GREM1 in PDAC was performed using analyzing high-throughput data in the database.

### Tumor immune estimation resource

Tumor Immune Estimation Resource (TIMER) (https://cistrome.shinyapps.io/timer/) is a user-friendly web server for systematic and comprehensive analysis of immune infiltration across various tumor types *via* inputting function-specific parameters (19). The immune infiltration of GREM1 in PDAC tissues was estimated using the TIMER database (Spearman’s correlation) in their different conditions (None, Tumor Purity, and Age).

### Statistical analysis

Statistical analysis and graphs were performed and plotted by SPSS v.25.0 (IBM Corp., Armonk, NY, USA) and GraphPad Prism 6.0 (La Jolla, CA, USA). Receiver operating characteristic (ROC) curves were conducted, and area under the curve (AUC), sensitivity, and specificity were calculated to compare the diagnostic value of GREM1 and other markers. Based on the ROC results, the cutoff of GREM1 was obtained to achieve the division of the low- and high-GREM1 groups. Meanwhile, different cutoff values were calculated by the X-tile program to explore the overall survival of the low- and high-GREM1 groups. Logistic regression analysis by SPSS software and Kaplan–Meier analysis by GraphPad Prism were performed in the two groups. Comparisons between two groups were conducted using a two-tailed Student’s t-test. For comparisons of three or more groups, the one-way ANOVA with post-hoc Dunnett’s test or Tukey’s test was utilized. Continuous data were presented as the mean ± SD and analyzed using Student’s t-tests. Statistical significance was indicated as a *p*-value <0.05.

## Results

### Aberrant transcriptional levels of GREM1 in human cancers

**Figure 1:**
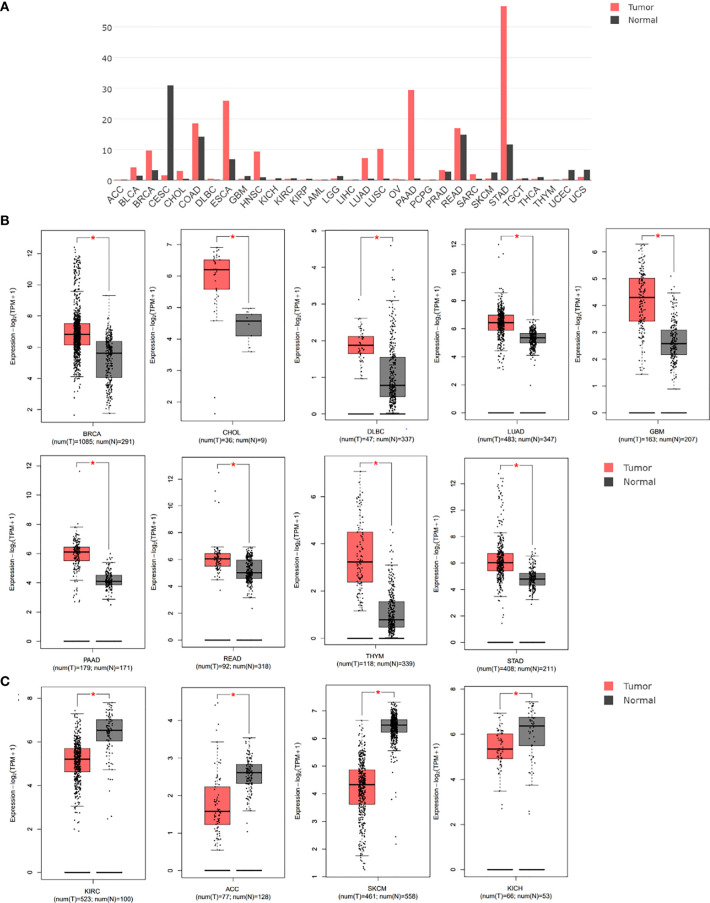
Gene expression differences of GREM1 were analyzed using the GEPIA database based on TCGA and GTEx databases. **(A)** Expression profile of GREM1 expression in 31 tumor types. **(B)** Higher expression of GREM1 in breast invasive carcinoma (BRCA), cholangial carcinoma (CHOL), lymphoid neoplasm diffuse large B-cell lymphoma (DLBC), lung adenocarcinoma (LUAD), glioblastoma multiforme (GBM), pancreatic ductal adenocarcinoma (PDAC, equal to PAAD used by bioinformatics databases), rectum adenocarcinoma (READ), thymoma (THYM), and stomach adenocarcinoma (STAD). **(C)** Lower expression of GREM1 in kidney renal clear cell carcinoma (KIRC), adrenocortical carcinoma (ACC), skin cutaneous melanoma (SKCM), and kidney chromophobe (KICH). GEPIA, Gene Expression Profiling Interactive Analysis; TCGA, The Cancer Genome Atlas; GTEx, Genotype-Tissue Expression. * means p-value < 0.05.

**Figure 2:**
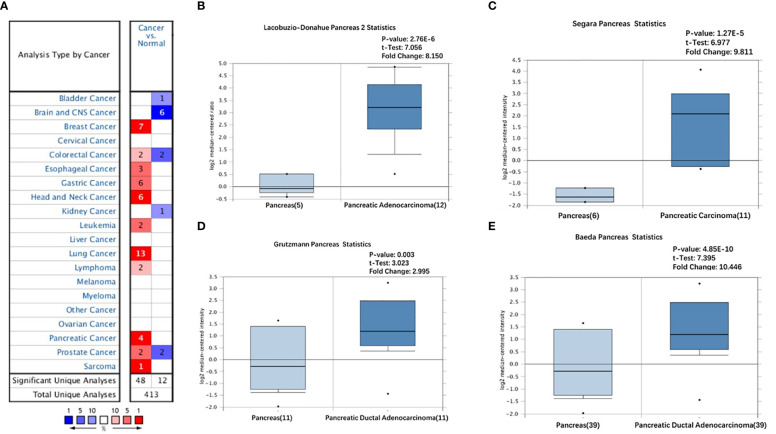
Overexpression of GREM1 in PDAC based on Oncomine database. **(A)** Different expression of GREM1 in multiple tumors (fold change > 2, *p*-value < 0.05). **(B–E)** The increase of GREM1 mRNA in four studies. PDAC, pancreatic ductal adenocarcinoma.

We initially analyzed the mRNA expression of GREM1 in human cancers using the database GEPIA. Its elevation in transcriptional level could be observed in multiple cancers, incorporating breast invasive carcinoma (BRCA), cholangial carcinoma (CHOL), lymphoid neoplasm diffuse large B-cell lymphoma (DLBC), lung adenocarcinoma (LUAD), glioblastoma multiforme (GBM), pancreatic ductal adenocarcinoma (PDAC), rectum adenocarcinoma (READ), thymoma (THYM), and stomach adenocarcinoma (STAD), whereas there were negative expressions in some neoplasms, including kidney renal clear cell carcinoma (KIRC), adrenocortical carcinoma (ACC), skin cutaneous melanoma (SKCM), and kidney chromophobe (KICH), compared with the corresponding normal tissues (**Figures 1A–C**). Notably, higher expression of GREM1 in PDAC tissues was reconfirmed in the Oncomine database (**Figure 2A**), and four patient cohort studies revealed its upregulation in PDAC (**Figures 2B–E**). Thus, the above results showed the differential expression of GREM1 between normal tissue and tumor, suggestive of an important regulatory role in tumor progression.

### GREM1 gene expression negatively correlated with poor prognosis in various cancer

**Figure 3:**
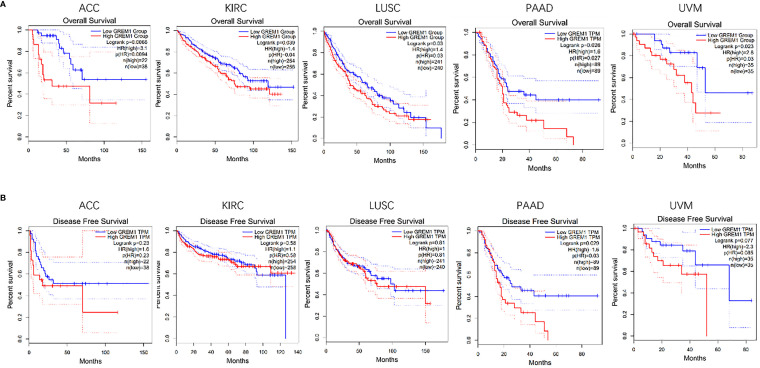
Survival analysis of GREM1 in multiple tumors was performed by Kaplan-Meier plotter. **(A)** OS in five tumors including PDAC. **(B)** DFS in five tumors including PDAC. OS, overall survival; PDAC, pancreatic ductal adenocarcinoma; DFS, disease-free survival.

By analyses of the GEPIA database, the upregulation of GREM1 gene expression in human cancers was of vital significance in the prognosis of multiple cancers. The analysis from the GEPIA database presented the poor OS in multiple tumors as the high expression of *GREM1* gene, such as the ACC, KIRC, lung squamous cell carcinoma (LUSC), PDAC, and uveal melanoma (UVM) (**Figure 3A**). Among these solid tumors, the difference in DFS was only PDAC (**Figure 3B**), predicting that the upregulation of GREM1 contributed to high relapse probability following treatment. These results manifested the potent capacity of GREM1 to forecast the prognosis of PDAC.

### Translational levels of GREM1 in pancreatic ductal adenocarcinoma

**Figure 4:**
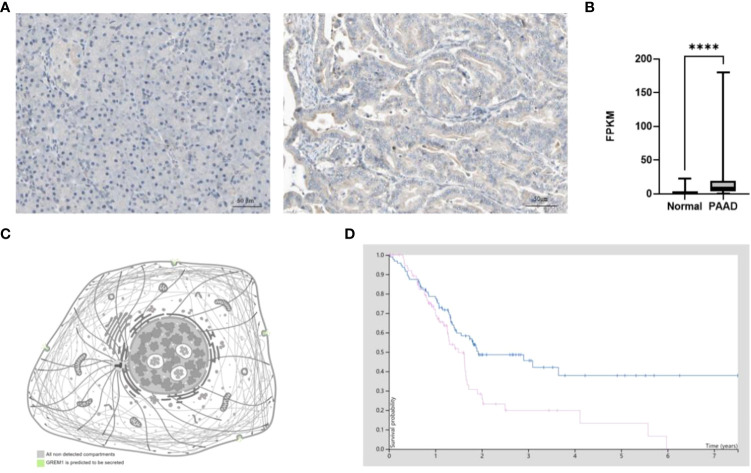
Protein level difference of GREM1 in PDAC based on HPA database. **(A)** Immunohistochemical staining of GREM1 in normal pancreas tissues and PDAC. **(B)** The difference in GREM1 mRNA transcription between normal pancreas tissues of 248 patient samples and PDAC of 176 patient samples from TCGA database. **(C)** GREM1 is secreted into the exocellular matrix. **(D)** Survival probability of PDAC patients with GREM1 overexpression (*p* = 0.011). PDAC, pancreatic ductal adenocarcinoma; HPA, Human Protein Atlas; TCGA, The Cancer Genome Atlas. **** means p-value < 0.0001.

GREM1 protein was investigated in PDAC by IHC staining, and the results found that GREM1 at the protein level was elevated in tumor tissues in contrast with normal pancreatic tissues (**Figure 4A**). From the results of IHC, GREM1 protein concentrated in interstitial space and executed its function in an exocellular environment. The protein level of GREM1 in PDAC was also higher than in the normal pancreas from the analyses of TCGA data (**Figure 4B**). According to the HPA database, GREM1 protein was reckoned as a type of secreted protein to exocellular stroma (**Figure 4C**), implicating that this message molecule might be involved in the signaling transmission and intercellular communication. Survival analysis revealed that patients with high-level GREM1 protein had shorter survival (**Figure 4D**). The results illuminated that GREM1 was overexpressed in PDAC and might promote PDAC progression.

### The pro-tumoral role of GREM1 correlated with stroma formation

**Figure 5:**
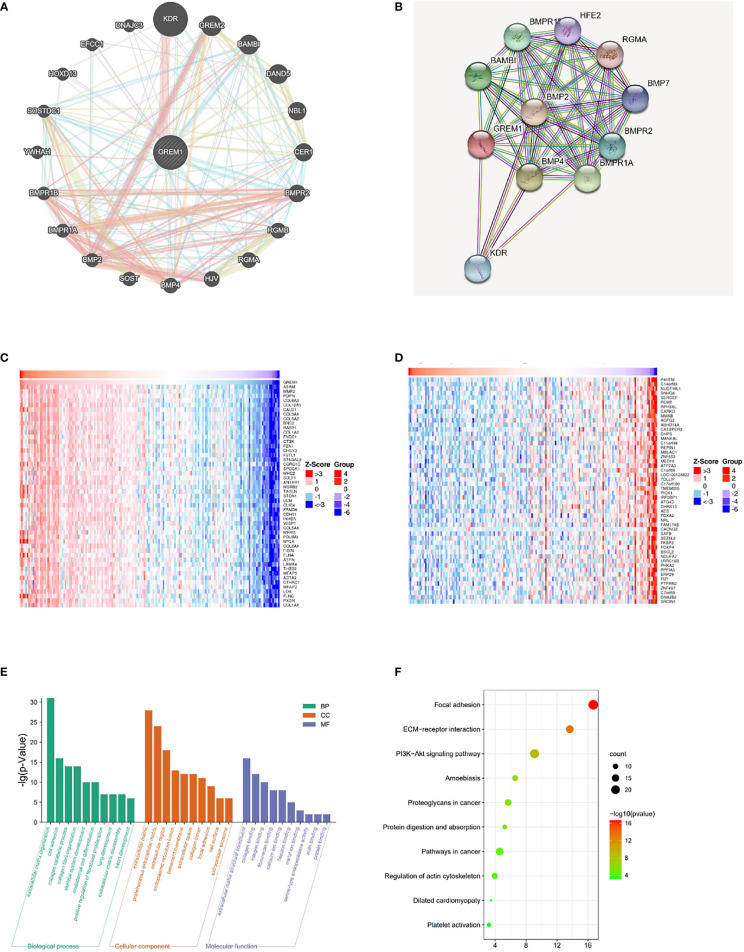
Related gene analysis of GREM1 and pathway function prediction in PDAC. **(A)** The gene–gene interaction network of GREM1 was constructed using GeneMANIA. **(B)** The PPI network of GREM1 was generated using STRING. **(C, D)** A heatmap shows the correlations between positively and negatively related significant genes in PDAC by LinkedOmics (Spearman’s correlation). **(E)** GO and **(F)** KEGG analyses for GREM1. PDAC, pancreatic ductal adenocarcinoma; PPI, protein–protein interaction; GO, gene ontology; KEGG, Kyoto Encyclopedia of Genes and Genomes.

The gene–gene interaction network for GREM1 in GeneMANIA exhibited 20 related functional genes, including KDR, GREM2, and BMP2 (**Figure 5A**). These molecules have been proved by previous studies as pro-tumoral factors in tumor progression (20). A PPI network of GREM1 through STRING analysis demonstrated the intimate relationship between GREM1 and BMP family, and its regulation could be implemented *via* a BMP-related pathway (**Figure 5B**). The results indicated that the function of GREM1 was linked to stroma formation, suggesting a significant role in tumor microenvironment constitution. The top 50 genes positively and negatively related to GREM1 in PDAC are shown in heatmaps (**Figures 5C, D**) (positively and negatively correlated gene lists are provided in **Supplementary Table 1**). Then, the top 200 genes positively related to GREM1 were used for GO and KEGG methods to predict the correlated signaling pathways and diverse biological functions (the top 200 positively correlated gene lists are provided in **Supplementary Table 2**; **Figures 5E, F**). The top 10 significant terms of BP, MF, and CC analytic results were obtained by the David database (**Figure 5E**). These data illustrated that GREM1 mainly acted in an exocellular environment and worked from cell to cell. In terms of BP, its function was associated with extracellular matrix organization, cell adhesion, and collagen catabolic. In terms of CC, the most correlated significant functions were extracellular matrix, proteinaceous extracellular matrix, and extracellular region. In terms of BP, its related functions were extracellular matrix structural constituent, collagen binding, and integrin binding. They all pointed to the regulation of exocellular matrix and were possibly engaged in exocellular signaling pathway transmission and integrate modulation of tumor external environment. These results revealed that GREM1 participated in exocellular environment constitution and it might play an important and extensive role in multiple biological effects, which was of great potential in the integrated modulation of the tumor microenvironment.

### Immune modulation roles of GREM1 in pancreatic ductal adenocarcinoma

**Figure 6:**
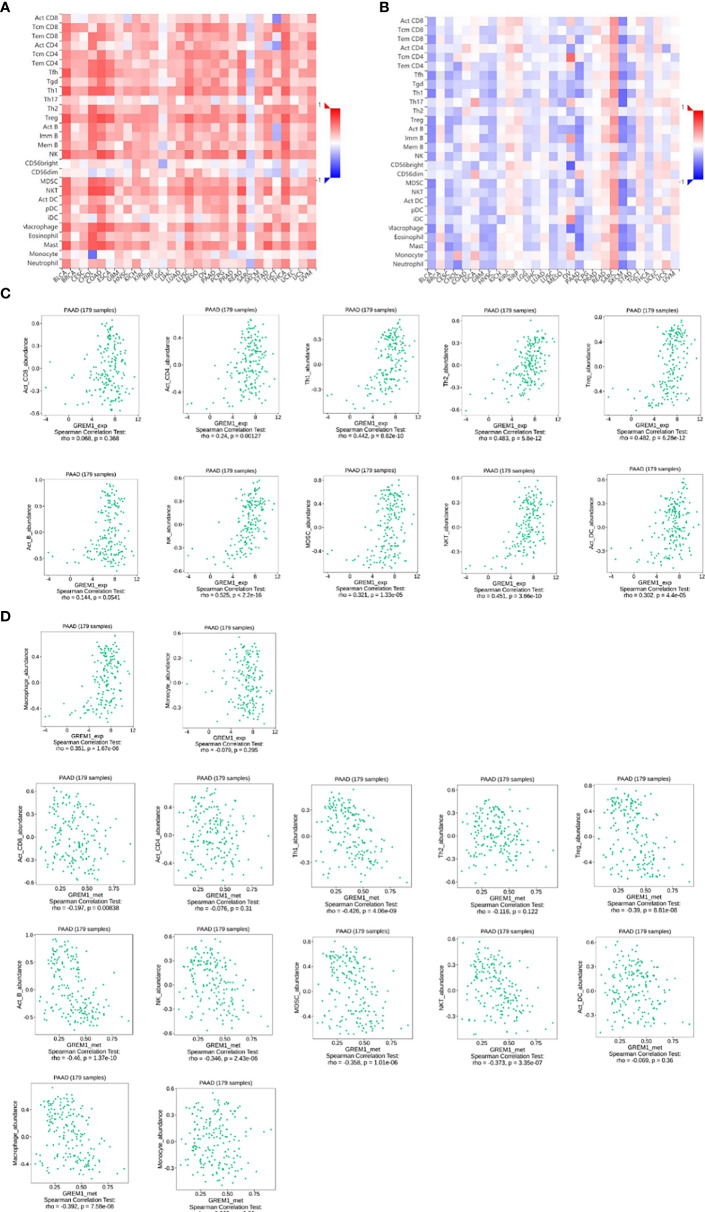
GREM1 overexpression regulates immune cell infiltration of PDAC. **(A)** Heatmap of immune cell infiltration in PDAC *via* TISID database. **(B)** Heatmap of immune cell infiltration in PDAC after GREM1 methylation. **(C)** Scatterplots of the correlations between GREM1 expression and immune cell infiltration and **(D)** GREM1 methylation and immune cell infiltration. PDAC, pancreatic ductal adenocarcinoma.

**Figure 7:**
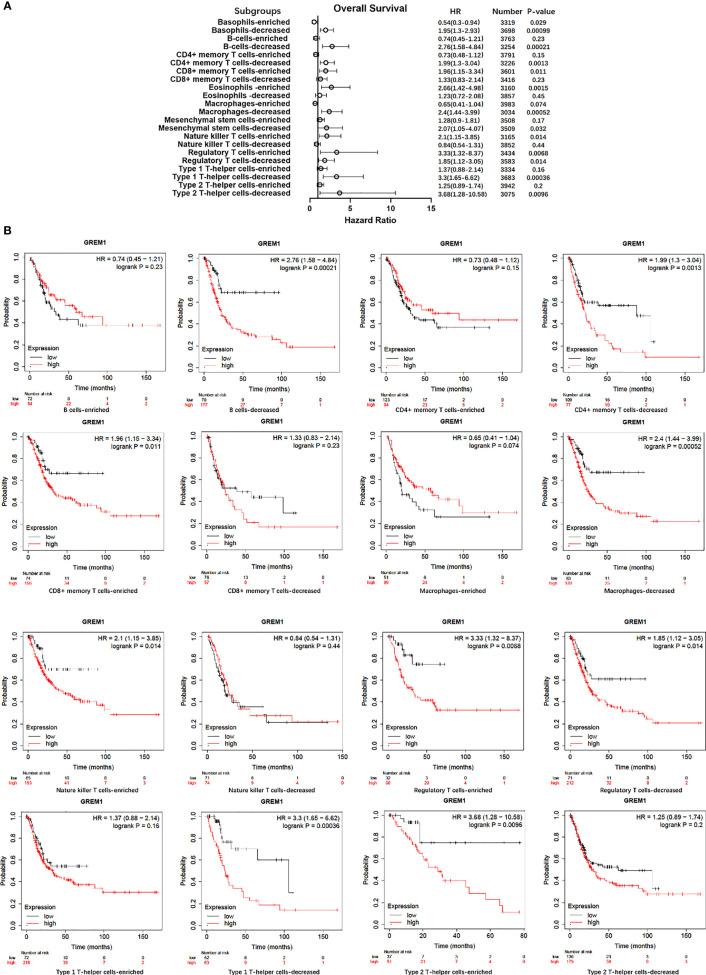
Survival analysis of immune cell infiltration in GREM1 high-expressed or low-expressed patients. **(A)** Forest plot for the total analytic data. **(B)** Survival analysis concerning GREM1 expression and immune cell infiltrations.

**Figure 8:**
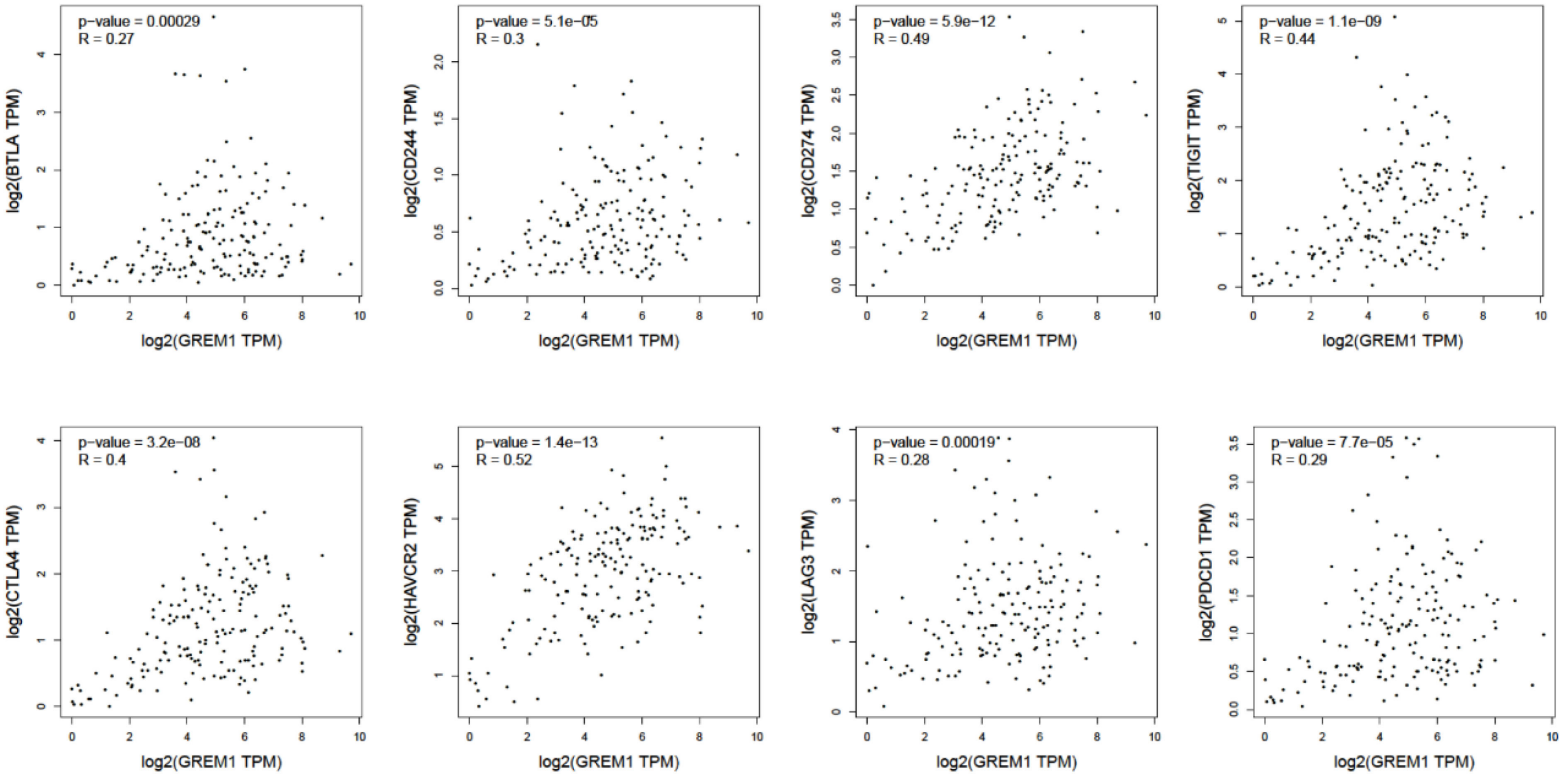
The positive relationship between GREM1 expression and several surface markers concerning T-cell exhaustion (BTLA, CD244, CD274, TIGIT, CTLA4, HAVCR2, LAG3, and PDCD1).

Immune cell infiltration in the tumor microenvironment of PDAC is exhibited in **Figure 6A**, showing the complicated alterations of immune components in PDAC. We observed that the immunosuppressive cells were enhanced by GREM1 expression, including T regulatory cells (Tregs), macrophages, myeloid-derived suppressor cells (MDSCs), CD4^+^ T cells, and Th2 cells (**Figure 6B**), in agreement with previous studies that reported that these cells were positively correlated with immunosuppression (21, 22). NK, NKT, and DC were positively related to GREM1 expression (**Figure 6B**), associated with tumor cell killing, but a recent study revealed the functional silence in the tumor microenvironment of PDAC and thus facilitated tumor progression. Therefore, the specific roles of these cells needed more investigation. Interestingly, methylation of GREM1 (GREM1 downregulation) induced the opposite results in these cells in **Figures 6B, D**, implying that low methylation of GREM1 might trigger immune cell infiltration. We further explored the infiltration of immune cells with the prognosis of PDAC. From the subgroup analysis of **Figures 7A, B**, the pro-tumor role of GREM1 could be embodied in CD8+ memory T cell, Treg, and Th2 cell enriched groups, and B cell, CD4+ memory T cell, macrophage, Treg, and Th1 decreased groups, which illustrated the prognostic prediction of GREM1 in such immune cells increased or decreased infiltration conditions. The correlation between GREM1 expression and a variety of T-cell subtypes was proved by the TIMER database (**Table 2**). T-cell exhaustion was detected in the tumor microenvironment of PDAC with GREM1 expression (**Figure 8**). A series of markers symbolized T-cell exhaustion, such as CTLA4, PD-1, PD-L1(CD244), LAG3, TIM3(HAVCR2), BTLA, 2B4(CD244), and TIGIT, were positively associated with GREM1 expression. These results demonstrated that GREM1 had potent immune modulation in PDAC and promoted tumor progression by sustaining immunosuppression.

**Table 2:** Correlation analysis between GREM1 and gene markers of different subgroups of T cells in PDAC by TIMER analysis.

Different groups	Gene markers	None		Tumor purity		Age	
		Cor	*p*	Cor	*p*	Cor	*p*
Th1	TBX21	0.238	**	0.203	**	0.238	**
	STAT4	0.196	**	0.197	**	0.185	*
	STAT1	0.434	***	0.395	***	0.432	***
	TNF	0.260	***	0.229	**	0.254	***
	IFNG	0.330	***	0.301	***	0.336	***
Th1-like	HAVCR2	0.475	***	0.431	***	0.471	***
	IFNG	0.330	***	0.301	***	0.336	***
	CXCR3	0.249	***	0.208	**	0.250	***
	BHLHE40	0.163	*	0.140	0.067	0.157	*
	CD4	0.414	***	0.364	***	0.409	***
Th2	STAT6	0.142	0.057	0.124	0.107	0.138	0.066
	STAT5A	0.337	***	0.305	***	0.335	***
Treg	FOXP3	0.436	***	0.395	***	0.431	***
	CCR8	0.481	***	0.443	***	0.477	***
	TGFB1	0.377	***	0.346	***	0.371	***
Resting Treg	FOXP3	0.436	***	0.395	***	0.431	***
	IL2RA	0.489	***	0.449	***	0.484	***
Effector Treg T cell	FOXP3	0.436	***	0.395	***	0.431	***
	CCR8	0.481	***	0.443	***	0.477	***
	TNFRSF9	0.512	***	0.479	***	0.508	***
Effector T cell	CX3CR1	0.042	0.6	-0.005	0.949	0.036	0.632
	FGFBP2	0.282	***	0.285	***	0.286	***
	FCGR3A	0.535	***	0.506	***	0.536	***
Naive T cell	CCR7	0.249	***	0.202	***	0.241	***
	SELL	0.260	***	0.204	***	0.256	***
Effector memory T cell	DUSP4	0.070	0.353	0.073	0.4	0.070	0.357
	GZMK	0.252	***	0.207	**	0.249	***
	GZMA	0.275	***	0.247	**	0.275	***
Resident memory T cell	CD69	0.356	***	0.314	***	0.350	***
	CXCR6	0.414	***	0.366	***	0.409	***
	MYADM	0.519	***	0.496	***	0.514	***
General memory T cell	CCR7	0.249	***	0.202	***	0.241	***
	SELL	0.260	***	0.204	***	0.256	***
	IL7R	0.503	***	0.466	***	0.498	***
Exhaustion T cell	HAVCR2	0.475	***	0.431	***	0.471	***
	LAG3	0.305	***	0.277	***	0.306	***
	CXCL13	0.276	***	0.230	**	0.273	***
	LAYN	0.735	***	0.717	***	0.733	***

Values are corrected by Tumor purity and Age.

PDAC, pancreatic ductal adenocarcinoma; TIMER, Tumor Immune Estimation Resource.

*p < 0.05, **p < 0.01, ***p < 0.001.

### Clinical significance of serum GREM1 in pancreatic ductal adenocarcinoma diagnosis and prognosis

**Figure 9:**
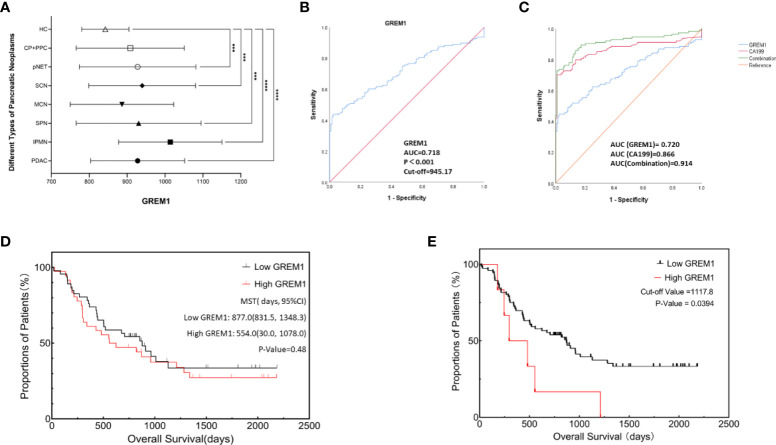
Diagnosis and prognosis analysis of serum GREM1 to explore clinical significance. **(A)** GREM1 levels in patient serum with different types of pancreatic neoplasms were detected and were all elevated, compared to the healthy control group (HCs, n = 132); these serum samples included pancreatic ductal adenocarcinoma (PDAC; n = 128), intraductal papillary mucinous neoplasm (IPMN; n = 39), pancreatic solid pseudopapillary neoplasm (SPN; n = 47), pancreatic neuroendocrine tumor (pNET; n = 54), serous cystadenoma (SCN; n = 31), mucinous cystadenoma (MCN; n = 26), chronic pancreatitis (CP; n = 11), pancreatic pseudocyst (PPC; n = 4), and healthy control (HCs; n = 132). **(B)** ROC analysis was performed for GREM1 in 260 cases of PDAC patients (AUC = 0.718, *p* < 0.001), and the cutoff value was calculated to be 945.17. **(C)** ROC analysis for GREM1, CA199, and their combinative diagnostic effect (combination value = GREM1 + CA199 * 0.44/0.09, 0.44, and 0.99 are the coefficient factors from logistic regression equation, AUC = 0.914 *p* < 0.001). **(D)** Survival curves from Kaplan–Meier analysis to compare the low- and high-GREM1 (*p* = 0.48). **(E)** Kaplan–Meier curve for overall survival of PDAC patients based on an optimal cutoff value calculated by X-tile program; OS of high-GREM1 group (n = 6) was significantly poorer than that of the low-GREM1 group (n = 76), *p* = 0.0394. ROC, receiver operating characteristic; AUC, area under the curve; CA199, carbohydrate antigen 199; OS, overall survival.#***p < 0.001, ****p < 0.0001.

Serum GREM1 levels in PDAC and other pancreatic neoplasms were obtained at varying degrees of elevation, compared to the HC group (**Figure 9A**, *p* < 0.05). We could not observe the significant difference between PDAC and other tumors. Accordingly, 128 cases of PDAC serum stepped into further investigation. Serum GREM1 of 260 PDAC patients underwent the ROC analysis, and the results showed its excellent diagnostic value, with an AUC of 0.718 (**Figure 9B**, *p* < 0.001). Based on the ROC result, the cutoff value was calculated as equal to 945.17 pg/ml. The combinative effect of serum GREM1 and CA199 was also equally evaluated, which presents the higher diagnostic value of CA199 allied with serum GREM1 (**Figure 9C**, *p* < 0.001).

PDAC patients were divided into the low-GREM1 (n = 72) and high-GREM1 (n = 56) groups by the cutoff value. Subsequently, several associated factors were analyzed by logistic regression (**Table 3**). Age, sex, smoking, drinking, hypertension, tumor locations, distant metastasis, and tumor stages did not exhibit a significant correlation with the two GREM1 groups. Diabetes was a negatively correlated factor (**Table 3**, *p* = 0.034). GREM1 was probably related to tumor growth, resulting from the positive correlation with tumor size (HR = 7.097, *p* = 0.032) and histopathological grades (HR = 2.898, *p* = 0.014). However, it was negatively paralleled with lymph node metastasis (HR = 0.149, *p* = 0.036). It suggested that GREM1 promoting stromal construction may contribute to the blockade of matrix degeneration and indicated its crucial role in tumor growth from another aspect. In addition, a survival analysis of 82 PDAC patients with radical resection surgery by the Kaplan–Meier method was performed to acquire exploration on the relationship between serum GREM1 and postoperative survival (**Figure 9D**, *p* = 0.48). The mean survival time of the low-GREM1 group was 877.0 days (831.5, 1,348.3), while that of the high-GREM1 group was 554.0 (30.0, 1,078.0) days (*p* = 0.48). Additionally, the X-tile program was used to explore the optimal cutoff value of overall survival time. As the cutoff value was equal to 1,117.8 pg/ml, the overall survival of the high-GREM1 group (n = 6) was significantly shorter than that of the low-GREM1 group (n = 76) (**Figure 9E**, *p* = 0.0394). Since sample capacity in our cohort was limited, its prognostic efficiency in PDAC patients was restricted. However, it proved the possibility of GREM1 predicting PDAC prognosis on the condition of enlarging patient volumes. Taken together, these results illustrated that serum GREM1 was a risk factor for PDAC and that its level in serum was an excellent potential marker for diagnosis of PDAC and a potential predictor for prognosis.

**Table 3:** Logistics analysis of GREM1 and correlated factors.

Items	Coefficient	*p*-Value	OR	95%CI	
				Lower limitation	Upper limitation
Age (<62/≥62)	0.127	0.722	1.135	0.565	2.284
Sex (male/female)	−0.193	0.590	0.824	0.408	1.666
Smoking (yes/no)	−0.281	0.542	0.755	0.306	1.861
Drinking (yes/no)	0.138	0.753	1.147	0.488	2.699
Hypertension (yes/no)	0.177	0.639	1.193	0.570	2.498
Diabetes (yes/no)	−0.811	0.034*	0.444	0.210	0.942
Tumor size (T1/T2/T3)	1.960	0.032*	7.097	1.188	42.406
Tumor location (head and neck/body and tail)	0.745	0.058	2.107	0.975	4.552
Histopathological grades (Grades 1–2/Grades 3–4)	1.064	0.014*	2.898	1.236	6.798
Lymph node metastasis (N0/N1/N2)	−1.906	0.036*	0.149	0.025	0.885
Distant metastasis (M0/M1)	−0.017	0.987	0.984	0.129	7.474
Tumor stage (1A-2A/2B-4)	19.871	1	–	–	–

*p < 0.05, means statistical significance.

## Discussion

GREM1, a pleiotropic regulator shuttling in fine-tuning BMP, takes charge of tissue development and organ morphology (9, 20). Researchers have focused on bone development, in recent years, and have witnessed the miraculous manipulation of tumor progression (13, 23). Consistent with our analytic data, elevations of GREM1 expression were visible in numerous solid tumors, such as lung cancer, kidney cancer, and gastric cancer. PDAC secured the augmentation of GREM1 expression to perform its all-around tumor promotion. As shown in our survival analysis, high expression of GREM1 in some tumors obtained the consequence of shorter OS and DFS. Additionally, we detected serum GREM1 level to uncover its excellent diagnostic and predictive potential for PDAC patients.

Previous studies have reported that GREM1 enhanced the proliferation, invasiveness, and metastasis of tumors through VEGFR2 and BMP-related pathways (20, 24). In GREM1-silencing cells, p53 phosphorylation and expression of its target gene p21 are enhanced to reduce cell survival *via* programmed death (25). Angiogenesis is regulated by GREM1 to elevate the microvessel density in pancreatic neuroendocrine tumors (24). From the results of HPA, GREM1 protein was predicted to be secreted to the exocellular matrix by the way of exocytosis. The environmental abundance of GREM1 increased in PDAC tissues more than in healthy pancreas tissues, which often predicted a worse prognosis, in agreement with our multiple survival analyses and the confirmation of our follow-up cohort study. Pathway prediction analyses offered the complicated interaction network formed by the downstream signaling pathways targeted. A significant role in extracellular matrix (ECM)–receptor interaction decides the stroma construction in tumor cells. A recent study has proposed the manipulation of MMP generation *via* activating signal transducer and activator of transcription 3 (STAT3) to catalyze the matrix degeneration and cellular disconnection, thus facilitating the metastasis of tumor cells (26). Desmoplastic activation has been linked to the upregulation of GREM1, which determines the rapid progression of tumors (27). Desmoplasia in PDAC is of vital importance, blocking anti-immunity and therapeutic drug delivery, which is regarded as a promoter of malignant progression (28). Combined with our findings, this seems to indicate that GREM1 may promote PDAC progression *via* regulating desmoplasia.

The tumor microenvironment embodies the harmony of tumor cells and the surroundings; the interaction between tumor cells and stroma allows them to reach a state of mutual accommodation. Due to the dense stroma and severe chronic inflammation in PDAC, the constitution of the tumor microenvironment is of vital significance for tumor cells survival; thus, the destruction degree of tumor microenvironment homeostasis determines the success or failure of combating tumor (29). Previous studies have pointed out that PDAC is characterized by the infiltration of immunosuppressive cells and the transformation of antitumor into pro-tumor immunity (30, 31). Distinct from a previous study on GREM1 in pancreatic tumors (24, 32), our analysis has reported characteristic immune cell infiltration featuring distinctive immunosuppressive properties in the pancreatic tumor microenvironment, so that the molecule GREM1 acting outside cell may modulate the immune structure and immune efficiency distribution to facilitate tumor progression. The previous study has also revealed the adverse prognostic factor in lung cancer, which similarly induces the infiltration of immunosuppressive cells (33). Characteristically, GREM1 induces massive infiltrations of immunosuppressive cells incorporating macrophages, Tregs, and MDSCs, repressing immune cells to identify and eliminate tumor cells (31). Poor prognosis along with GREM1 overexpression correlated with several immune cell infiltration subgroups, indicative of some specific immune structure that can make sense to GREM1 expression in PDAC. Martin *et al.* have proposed that the combination of low budding, low stromal FOXP3 counts, presence of TLTs, and absence of CDKN2A mutations confers a significant survival advantage in patients with PDAC (34). Here, the direct proportion function of GREM1 expression and FOXP3^+^ Tregs can be observed. Interestingly, the T-cell cluster is investigated in PDAC, presenting a declining tendency in T-cell activation. The upregulation of GREM1 predicts the T-cell exhaustion, a rise in relevant inhibitory surface receptor-like CTLA4, PD-1, PD-L1(CD244), LAG3, TIM3(HAVCR2), BTLA, 2B4(CD244), and TIGIT (35), illuminating the state of T-cell exhaustion. Taken together, GREM1 maintains the immunosuppressive tumor microenvironment, thus promoting PDAC progression.

Furthermore, preliminary verifications were carried out to explore the diagnostic and prognostic values of GREM1 in serum. Concerning the bioinformatics analysis results, serum GREM1 level significantly increased in the PDAC group compared to the HC group, as well as different elevations in assorted pancreatic neoplasms, especially IPMN. Nevertheless, we cannot observe the significant difference among different types of pancreatic diseases due to the limitation of sample quantity. Exactly, serum GREM1 has a good diagnostic value, and its alliance enhances the diagnostic effect of CA199, the most commonly used PDAC diagnostic marker in clinical practice. Interestingly, the increasing level of serum GREM1 occurred in bigger tumor diameters and advanced histopathological grades, as the primary result of GREM1 representing a formidable stromal factor and functioning as stroma modulation. Ultimately, the negative impact on survival of serum GREM1 was visible, despite the disability to statistical significance due to sample restriction, equally identical to multiple survival analyses, which should be further verified by enrolling more PDAC patients.

In summary, GREM1 is significantly upregulated in multiple cancers, including PDAC, which indicates a faster relapse and shorter survival for patients with PDAC. Its pro-tumoral effects in PDAC are pleiotropic, predominantly in promoting stroma formation *via* desmoplasia and inducing immunosuppression in the tumor microenvironment. Our bioinformatics analysis offers a preliminary exploration and discussion on the function of GREM1 in PDAC, and our clinical data further demonstrate the good diagnostic potential of serum GREM1, especially in combination with CA199, which is expected to be a potential candidate for diagnosis and therapy of PDAC.

## Data availability statement

The original contributions presented in the study are included in the article/**Supplementary Material**. Further inquiries can be directed to the corresponding authors.

## Author contributions

SY, YZ, QFL, and QL conceived this study, analyzed the data, and drafted the manuscript. QFL reviewed and revised the manuscript. MC, MW, JG, and YH collected the data and reviewed the manuscript. QFL and QL were responsible for supervision and project administration. All authors contributed to the article and approved the submitted version.

## Funding

This work was supported by the National Natural Science Foundation of China (82172765, 81872501), CAMS Innovation Fund for Medical Sciences (CIFMS,2021-I2M-1-002), Beijing Natural Science Foundation (7172177), and Youth Foundation of Peking Union Medical College Hospital (pumch201911866).

## Conflict of interest

The authors declare that the research was conducted in the absence of any commercial or financial relationships that could be construed as a potential conflict of interest.

## Publisher’s note

All claims expressed in this article are solely those of the authors and do not necessarily represent those of their affiliated organizations, or those of the publisher, the editors and the reviewers. Any product that may be evaluated in this article, or claim that may be made by its manufacturer, is not guaranteed or endorsed by the publisher.
